# Quality of life score as a prognosticator for pharyngeal cancer patients treated with radiotherapy

**DOI:** 10.1038/s41598-022-06441-y

**Published:** 2022-02-11

**Authors:** Hiromichi Ishiyama, Shogo Kawakami, Akane Sekiguchi, Takuro Kainuma, Shunsuke Miyamoto, Taku Yamashita, Masahiro Nakano

**Affiliations:** 1 Department of Radiation Oncology, Kitasato University School of Medicine, 1-15-1 Kitasato, Minamiku, Sagamihara, Japan; 2 Department of Otorhinolaryngology and Head and Neck Surgery, Kitasato University School of Medicine, 1-15-1 Kitasato, Minamiku, Sagamihara, Japan

**Keywords:** Oncology, Risk factors, Outcomes research

## Abstract

The purpose of this study was to evaluate the prognostic value of quality of life (QOL) scores acquired not only pre-treatment, but also 1 month after treatment for locoregional control (LRC), distant metastasis-free survival (DMFS), and overall survival (OS) in patients with pharyngeal cancer treated using radiotherapy. Data for 102 patients with naso-, oro-, or hypo-pharyngeal cancer treated between December 2008 and September 2017 were retrospectively analyzed. About 90% of the patients were male. The European Organization for Research and Treatment of Cancer (EORTC) Quality of Life Questionnaire (QLQ-C30) was used for QOL assessments. Associations between QLQ-C30 scores before and 1 month after treatment and outcomes including LRC, DMFS, and OS were analyzed using Cox proportional hazard models. Median follow-up was 37 months (range, 5–117 months). Three-year LRC, DMFS, and OS rates were 77.8%, 60.0%, and 66.5%, respectively. Pre-treatment emotional functioning and diarrhea at 1 month after treatment were identified as significant predictors of LRC. Pre-treatment global QOL and diarrhea at 1 month after treatment were detected as significant predictors of DMFS. Pre-treatment emotional functioning, pre-treatment appetite loss, and diarrhea at 1 month after treatment were detected as significant predictors of OS. Diarrhea at 1 month after treatment was the most powerful QOL variable for predicting LRC, DMFS and OS. Our study revealed that several QOL scores not only before treatment but also 1 month after treatment correlated with LRC, DMFS and OS. In particular, the diarrhea domain of QOL at 1 month after treatment offered the most powerful prognosticator for pharyngeal cancer patients treated with radiotherapy.

## Introduction

Quality of life (QOL) scores reported by patients themselves are well-known prognosticators for cancer patients. Even when limited to patients with head and neck cancer, many studies have revealed that pre-treatment QOL scores correlate with loco-regional control (LRC), distant metastasis-free survival (DMFS), and overall survival (OS)^1–22^.

However, no definitive QOL domain correlating with all these outcomes has previously been identified, although various domains have been detected as significant prognosticators in previous investigations. The following factors seem to contribute to conflicts and ambiguity in the results: (1) cancers with significantly different prognoses have all been included together; (2) patients treated with surgery, radiotherapy, and chemotherapy have all been included together; and (3) no unified QOL scoring tool has been applied. Therefore, pre-treatment QOL scores do not seem to have been widely used as prognosticators in clinical practice.

Another challenge with the use of pre-treatment QOL scores is that little room remains to modify the main treatment, even if the score suggests a need for more- or less-intensive treatment. From a clinical practice perspective, however, determination of QOL just after treatment might represent a good candidate as an intervention target, allowing opportunities for preventive countermeasures for affected QOL domains.

In this study, therefore, we selected only patients with naso-, oro-, or hypopharyngeal cancer treated with radiotherapy. We then assessed correlations between LRC, DMFS, OS and QOL not only pre-treatment, but also 1 month after treatment.

## Materials and methods

### Patients

The Kitasato University Hospital institutional review board approved this observational study (approval no. B12-27). This study was conducted in accordance with the Declaration of Helsinki. Data from 102 patients with pharyngeal cancer treated between December 2008 and September 2017 were retrospectively analyzed. All patients provided written informed consent before the QOL survey. Patient and treatment characteristics are summarized in Table 1. The majority of patients were male (90%). Seventy-five patients (74%) had locally advanced disease (Stage III or IV) and 86 patients (84%) received a combination of radiotherapy and concurrent chemotherapy. Patients who received neoadjuvant chemotherapy were excluded, because the QOL survey was conducted just before radiotherapy, but not before chemotherapy.

**Table 1:** Patient characteristics.

Age, years	68 (24–89)
**Sex**	
Male	91
Female	11
**Tumor site**	
Nasopharynx	20
Oropharynx	36
Hypopharynx	46
**Stage**	
I	3
II	23
III	24
IV	52
**PS**	
0	58
1	40
2	2
NA	2
**Concurrent chemotherapy**	
Yes	86
No	16
**Radiotherapy technique**	
IMRT	80
3D-CRT	22
Total dose, Gy	60 (46–70)

Values are number or median (range).

*IMRT* intensity-modulated radiotherapy, *3D-CRT* 3-dimensional conformal radiotherapy.

### Treatment

Radiotherapy was delivered using a conventional linac system (MHCL-15TP; Mitsubishi, Tokyo, Japan) or tomotherapy system (Tomotherapy; Accuray, Sunnyvale, CA, USA). All patients were immobilized with a face mask during planning computed tomography acquisition (slice thickness, 1.25–2.5 mm) and in all treatment sessions. For patients treated with intensity-modulated radiotherapy (IMRT), planning target volume (PTV)1 was defined as the initial target volume and included the primary tumor, lymph nodes known to contain metastases, and bilateral neck node levels considered at risk of microscopic disease with a 5-mm setup margin in all directions. PTV2 was defined as the boost target volume and included the primary tumor and lymph nodes known to contain metastases with the same setup margin. In the majority of patients, PTV1 was treated with 40 Gy in 20 daily fractions and PTV2 with 60 or 66 Gy in 30 or 33 daily fractions with a two-step technique. Five patients with nasopharyngeal cancer were treated using a simultaneous integrated boost technique with 54 Gy to PTV1, 60 Gy to the high-risk area of PTV1, and 66 Gy to PTV2. Sixty-seven of 80 patients (84%) treated with IMRT received concurrent chemotherapy.

For patients treated with 3-dimensional conformal radiotherapy (3D-CRT), the basic concept of the target was the same as that with IMRT. In the majority of patients, the initial target area including the primary tumor, lymph nodes known to contain metastases, and bilateral neck node levels considered at risk of microscopic disease was treated with 40 Gy in 20 daily fractions and the boost area including the primary tumor and metastatic lymph-node was treated with 60 or 66 Gy in 30 or 33 daily fractions. Nineteen of 22 patients (86%) treated with 3DCRT received concurrent chemotherapy.

### QOL assessment

The European Organization for Research and Treatment of Cancer (EORTC) Quality of Life Questionnaire (QLQ-C30) was used for QOL assessments. This questionnaire includes a global health status scale, five functional scales, and nine symptom scales. In this study, questionnaires filled out before (n = 102) and 1 month after (n = 102) completing radiotherapy were used. According to the EORTC scoring procedure, all scales of the questionnaire were converted into scores ranging from 0 to 100 points. A higher score for a global health status or functional scale denotes a higher level of global health status or functioning, whereas a higher score on a symptom scale denotes more severe symptoms.

### Statistical analysis

First, associations between LRC, DMFS, OS and clinical factors such as clinical stage based on International Union Against Cancer 7th edition, baseline performance status (PS), age, total radiation dose, tumor location, combined chemotherapy, sex, smoking index, and alcohol habits were analyzed using univariate analysis.

Second, associations between each QLQ-C30 score and outcomes were analyzed using multivariate analysis to control for clinical variables detected in univariate analyses. Hazard ratios (HRs) were calculated for every 10-point difference in scores on QLQ-C30 items^23^.

Cox proportional hazard modeling was used for uni- and multivariate analyses. All statistical analyses were performed using R version 3.5.1 software (R Foundation, Vienna, Austria). Values of p < 0.05 were considered statistically significant.

### Ethics approval and consent to participate

This retrospective study was approved by our institutional review board (approval no. B12-27).

### Consent for publication

All tables and figures contain anonymized patient data from which a particular patient cannot be identified.

## Results

### Univariate analyses of clinical variables

Median follow-up was 37 months (range, 5–117 months). Locoregional failure and distant metastases were seen in 20 patients (19.6%) each. Twenty-six patients (25.4%) died of pharyngeal cancer, 7 (6.9%) of other medical diseases, and 2 (2.0%) of unknown causes. Three-year LRC, DMFS, and OS rates were 77.8%, 60.0%, and 66.5%, respectively. Table 2 shows the results of univariate analyses of clinical variables. Tumor location was detected as a significant predictor of LRC. Clinical stage, PS, and smoking index were detected as significant predictors of DMFS and OS. As a result, tumor location, smoking index, clinical stage, and PS were used in multivariate models for QOL scores.

**Table 2:** Univariate analyses of clinical variables.

Variable	LRC				DMFS				OS			
	HR	95% CI		p value	HR	95% CI		p value	HR	95% CI		p value
**Clinical stage**	0.989	0.607	1.610	0.965	1.999	1.271	3.144	**0.003**	2.478	1.432	4.290	**0.001**
PS	1.516	0.672	3.421	0.316	1.905	1.084	3.349	**0.025**	2.653	1.457	4.830	**0.001**
Age	1.008	0.969	1.048	0.698	1.008	0.980	1.037	0.569	0.999	0.971	1.029	0.967
Total dose	0.994	0.880	1.123	0.922	1.013	0.929	1.105	0.770	1.019	0.927	1.119	0.702
**Tumor location**												
Hypo	Ref				Ref				Ref			
Oro	0.264	0.075	0.928	**0.038**	0.489	0.239	1.001	0.050	0.696	0.331	1.465	0.340
Naso	0.631	0.206	1.935	0.420	0.397	0.151	1.042	0.061	0.552	0.206	1.483	0.239
**Chemoradiotherapy**												
No	Ref				Ref				Ref			
Yes	0.507	0.184	1.397	0.189	0.960	0.403	2.289	0.927	1.822	0.557	5.956	0.321
**Sex**												
Female	Ref				Ref				Ref			
Male	1.144	0.265	4.930	0.857	1.632	0.503	5.296	0.415	2.122	0.509	8.850	0.302
Smoking index	1.000	1.000	1.001	0.146	1.001	1.000	1.001	**0.007**	1.001	1.000	1.001	**0.006**
Alcohol (drinks/day)	0.968	0.870	1.078	0.556	0.991	0.923	1.064	0.807	1.003	0.930	1.082	0.938

*PS* performance status, *LRC* locoregional control, *DMFS* distant metastasis-free survival, *OS* overall survival.

Significant values are in bold.

### Multivariate analyses of QOL and clinical variables

Each QOL variable was separately analyzed for LRC, DMFS, and OS in the multivariate analyses, controlling for clinical variables including clinical stage, PS, tumor location, and smoking index.

Table 3 shows the results for QOL variables after controlling for clinical variables. Pre-treatment emotional functioning and diarrhea at 1 month after treatment remained as significant predictors of LRC. Pre-treatment global QOL and diarrhea at 1 month after treatment were detected as significant predictors of DMFS. Pre-treatment emotional functioning, pre-treatment appetite loss, and diarrhea at 1 month after treatment remained as significant predictors of OS. Diarrhea at 1 month after treatment was thus the most powerful QOL variable for predicting LRC, DMFS and OS.

**Table 3:** Multivariate analysis QOL scores for LRC, DMFS, and OS.

Variable	LRC				DMFS				OS			
	HR	95% CI		p value	HR	95% CI		p value	HR	95% CI		p value
**Before treatment**												
Global QOL	0.890847	0.7282	1.0898	0.2611	0.861	0.751	0.989	**0.034**	0.864	0.744	1.002	0.054
Physical functioning	0.823	0.646	1.049	0.116	0.906	0.736	1.115	1.115	0.897	0.714	1.128	0.352
Role functioning	0.875	0.750	1.021	0.091	0.936	0.831	1.053	0.272	0.908	0.798	1.032	0.139
Emotional functioning	0.804	0.675	0.956	**0.014**	0.900	0.792	1.023	0.107	0.874	0.765	0.998	**0.047**
Cognitive functioning	0.941	0.737	1.201	0.625	0.932	0.792	1.097	0.399	0.918	0.775	1.086	0.318
Social functioning	0.861	0.727	1.020	0.084	0.892	0.788	1.011	0.074	0.881	0.772	1.005	0.059
Fatigue	1.143	0.915	1.429	0.238	1.126	0.976	1.298	0.103	1.159	0.994	1.350	0.060
Nausea/vomiting	0.844	0.438	1.628	0.613	1.139	0.849	1.529	0.387	1.082	0.786	1.490	0.628
Pain	1.115	0.934	1.332	0.229	1.115	0.982	1.267	0.093	1.138	0.992	1.306	0.065
Dyspnea	1.168	0.946	1.442	0.148	0.999	0.835	1.194	0.989	0.983	0.817	1.184	0.858
Insomnia	1.110	0.946	1.303	0.201	1.037	0.921	1.167	0.550	1.017	0.892	1.160	0.799
Appetite loss	1.070	0.911	1.258	0.409	1.108	0.996	1.232	0.060	1.156	1.029	1.299	**0.015**
Constipation	1.032	0.805	1.322	0.806	1.132	0.976	1.312	0.101	1.117	0.950	1.313	0.180
Diarrhea	0.914	0.658	1.270	0.591	1.096	0.918	1.309	0.313	1.138	0.944	1.373	0.174
**1 month after treatment**												
Global QOL	1.016	0.859	1.201	0.857	0.899	0.799	1.011	0.076	0.930	0.824	1.049	0.237
Physical functioning	0.956	0.821	1.114	0.566	0.935	0.843	1.038	0.207	0.906	0.814	1.009	0.071
Role functioning	0.983	0.856	1.127	0.802	0.968	0.882	1.063	0.499	0.929	0.840	1.027	0.148
Emotional functioning	0.969	0.856	1.096	0.611	0.943	0.866	1.027	0.179	0.961	0.881	1.049	0.378
Cognitive functioning	0.968	0.856	1.094	0.603	0.931	0.856	1.013	0.099	0.957	0.877	1.044	0.320
Social functioning	0.990	0.868	1.128	0.875	0.956	0.873	1.047	0.333	0.967	0.883	1.059	0.468
Fatigue	0.966	0.793	1.177	0.734	0.966	0.843	1.107	0.618	0.982	0.850	1.134	0.804
Nausea/vomiting	1.070	0.772	1.483	0.686	1.032	0.805	1.324	0.803	1.073	0.827	1.391	0.597
Pain	1.044	0.859	1.269	0.668	0.925	0.789	1.084	0.336	0.953	0.808	1.123	0.563
Dyspnea	0.980	0.793	1.209	0.847	0.982	0.854	1.129	0.800	0.969	0.832	1.128	0.685
Insomnia	0.958	0.817	1.124	0.597	0.992	0.891	1.105	0.880	0.989	0.881	1.111	0.857
Appetite loss	0.994	0.868	1.137	0.926	0.989	0.901	1.087	0.824	1.004	0.908	1.110	0.937
Constipation	0.859	0.681	1.084	0.201	0.964	0.828	1.123	0.640	0.908	0.764	1.080	0.277
Diarrhea	1.324	1.079	1.626	**0.007**	1.304	1.121	1.516	**0.001**	1.424	1.212	1.673	**0.000**

Each QOL variable was analyzed separtely in the model, adjusting for clinical stage, PS, tumor location, and smoking index.

HR measures correspond to 10-point increments in QLQ-C30 scores.

Abbreviations are the same as in Table 2.

Significant values are in bold.

**Figure 1:**
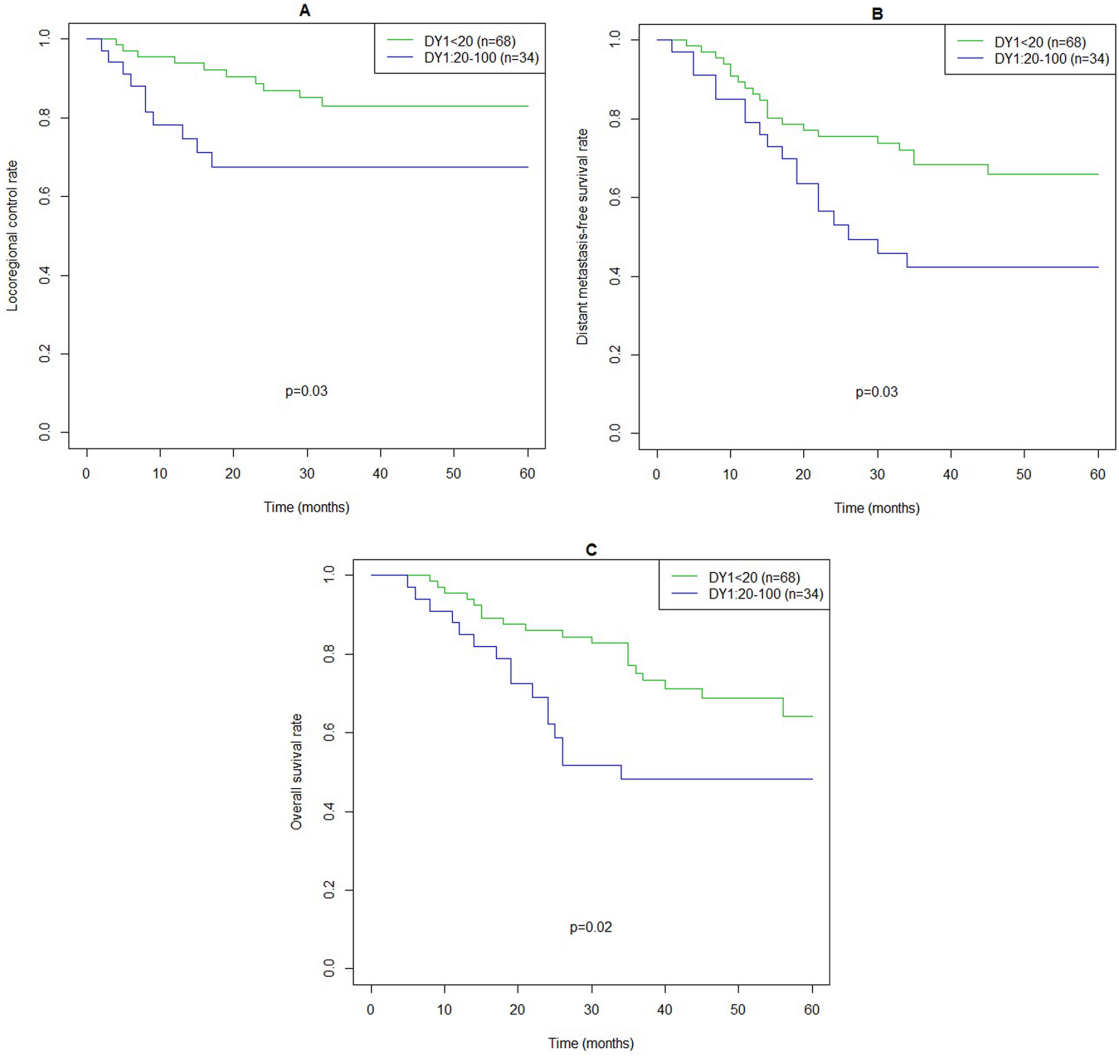
Locoregional control rate (**A**), distant metastasis-free survival rate (**B**) and overall survival rate (**C**) based on diarrhea score at 1 month after treatment. DY1: score for diarrhea at 1 month after treatment.

Table 4 shows multivariate models of clinical variables with or without diarrhea at 1 month after treatment for LRC, DMFS and OS. When diarrhea at 1 month after treatment was entered into the model, the likelihood ratio test statistic changed from 7.28 (χ^2^, df = 5) to 13.64 (χ^2^, df = 6) for LRC, from 27.65 (χ^2^, df = 5) to 37.87 (χ^2^, df = 6) for DMFS, and from 27.18 (χ^2^, df = 5) to 42.84 (χ^2^, df = 6) for OS. Figure 1 shows comparisons of LRC, DMFS and OS based on diarrhea at 1 month after treatment.

**Table 4:** Multivariate analysis of clincal variables and diahhrea for LRC and OS.

Variable	LRC				DMFS				OS			
	HR	95% CI		p value	HR	95% CI		p value	HR	95% CI		p value
Clinical stage	1.119	0.672	1.865	0.665	2.175	1.355	3.491	**0.001**	2.238	1.296	3.866	**0.004**
PS	1.392	0.598	3.240	0.443	1.444	0.783	2.664	0.240	2.059	1.065	3.981	**0.032**
**Tumor location**												
Hypo	Ref				Ref				Ref			
Oro	0.293014	0.082	1.048	0.059	0.470	0.226	0.977	**0.043**	0.743	0.348	1.587	0.443
Naso	0.724	0.231	2.268	0.579	0.460	0.174	1.221	0.119	0.719	0.265	1.953	0.518
Smoking index	1.000	1.000	1.001	0.261	1.001	1.000	1.001	**0.013**	1.001	1.000	1.001	**0.027**
Clinical stage	1.130	0.673	1.898	0.645	2.347	1.433	3.844	**0.001**	2.426	1.369	4.299	**0.002**
PS	1.435	0.600	3.432	0.417	1.516	0.809	2.843	0.194	2.430	1.221	4.838	**0.012**
**Tumor location**												
Hypo	Ref				Ref							
Oro	0.281	0.078	1.008	0.052	0.386	0.182	0.816	0.013	0.553	0.254	1.203	0.135
Naso	0.968	0.294	3.190	0.958	0.597	0.221	1.615	0.310	0.983	0.353	2.738	0.975
Smoking index	1.000	1.000	1.001	0.304	1.000	1.000	1.001	0.052	1.000	1.000	1.001	**0.112**
Diarrhea at 1 month	1.324	1.079	1.625	**0.007**	1.304	1.121	1.516	**0.001**	1.422	1.210	1.671	**0.000**

Abbreviations are the same as in Table 2.

Significant values are in bold.

## Discussion

Our study revealed several QOL scores from pretreatment and 1 month after treatment correlated with LRC, DMFS and OS for pharyngeal cancer patients treated using radiotherapy with or without chemotherapy. In particular, the diarrhea domain at 1 month after treatment was the most powerful indicator, and markedly increased the likelihood ratio test statistic of models.

Although the mechanisms by which diarrhea is associated with significant decreases in survival remain unclear, some hypotheses can be suggested. Diarrhea can be caused by radiotherapy, chemotherapy, and infectious diseases. In our study, however, radiotherapy was not basically correlated with diarrhea because only the head and neck region was irradiated. The main cause of diarrhea in our study population thus seems likely to have been chemotherapy.

Some chemotherapeutic agents, such as 5-fluorouracil, are well known to cause diarrhea^24^. Sixty-two patients in our study population received 5-fluorouracil and S-1 combined with cisplatin. However, receiving these drugs was not a significant prognosticator for any endpoints in the ad-hoc analysis (data not shown).

Interestingly, our study revealed that not only OS, but also LRC and DMFS correlated with the presence of diarrhea at 1 month after treatment. Some form of immune deficiency might thus be involved in these results. Although limited to laboratory animal studies, some evidence suggests that microbiota might contribute to anti-cancer treatment, with Maroof et al. reporting a significant tumor-suppression effect of *Lactobacillus acidophilus*, part of the intestinal flora, in an in-vivo model of breast cancer^25^. They also reported significant immunomodulatory effects from the intestinal flora. Gui et al. reported that destruction of the host intestinal flora by antibiotic drugs significantly reduced the treatment effects of cisplatin in a mouse model of lung cancer. Conversely, mice treated with cisplatin while receiving supplemental *Lactobacillus* bacteria showed increased tumor shrinkage and longer survival^26^. Such animal experiments suggest that the intestinal microflora might affect host immunity not only locally at the mucosal level, but also systemically. Preventive measures such as lactobacillus administration thus might prove effective for pharyngeal cancer patients.

Supporting our findings, Yang et al. reported that pre-treatment emotional sub-score correlated with disease-free survival and OS, although they used the MD Anderson dysphagia inventory as a measure of QOL^7^. Osthus et al. reported that pre-treatment appetite loss correlated with OS based on EORCT-C30 assessment^9^. In addition, Chen et al.^1^ and Truong et al.^4^ reported that pre-treatment overall QOL correlated with DMFS based on University of Washington QOL and Functional Assessment of Cancer Therapy-General, respectively. Therefore, regarding emotional state, appetite loss and global QOL, our results were compatible with some previous studies, although these domains were not as strongly associated with outcomes as diarrhea in our study.

One of the appealing points in the present study was the limitation of tumor location to naso-, oro-, and hypopharyngeal cancers. Although oropharyngeal caner showed significantly better outcomes for LRC compared to other tumor locations, no significant differences in DMFS and OS were apparent. Another appealing point of our study was that patients were only treated with radiotherapy with or without chemotherapy. The specific QOL domains suggested by our study might thus be reliable for pharyngeal cancer patients treated with radiotherapy, as they were derived from more homogeneous data than findings from previous studies.

Because this was a retrospective analysis, several limitations should be considered. First, as collected items were limited, other factors might correlate with LRC, DMFS, or OS. Second, patients with other sites of head and neck cancer, such as laryngeal cancer, might show result differing from those in our study. Third, because only the EORTC-C30 was used in our study, the results might not be the same as those from other QOL score systems. However, as diarrhea was clinically assessable, our results appear useful for any patient.

In conclusion, our study revealed that several QOL scores not only before treatment, but also 1 month after treatment correlated with LRC, DMFS and OS. Most importantly, the diarrhea domain of QOL at 1 month after treatment offered the most powerful prognosticator for pharyngeal cancer patients treated with radiotherapy.

## Data Availability

The datasets generated and/or analyzed during the current study are not publicly available, since the participants did not consent to the sharing of data with third parties.

## Abbreviations

QOL: Quality of life
LRC: Locoregional control
DMFS: Distant metastasis-free survival
OS: Overall survival
EORTC: The European Organization for Research and Treatment of Cancer
QLQ-C30: Quality of Life Questionnaire
PS: Performance status
HR: Hazard ratio
3D-CRT: 3-Dimensional conformal radiotherapy
IMRT: Intensity-modulated radiotherapy
PTV: Planning target volume
